# New Insights into the Prevalence of *Dirofilaria immitis* in Hungary

**DOI:** 10.3390/ani15091198

**Published:** 2025-04-23

**Authors:** Ákos Jerzsele, Dóra Kovács, Péter Fábián, Péter Fehérvári, Bettina Paszerbovics, Krisztina Bali, Eszter Kaszab, Nóra Mayer, Zita Karancsi

**Affiliations:** 1 Department of Pharmacology and Toxicology, University of Veterinary Medicine Budapest, 1078 Budapest, Hungary; jerzsele.akos@univet.hu (Á.J.); fabian.peti99@gmail.com (P.F.); karancsi.zita@univet.hu (Z.K.); 2 National Laboratory of Infectious Animal Diseases, Antimicrobial Resistance, Veterinary Public Health and Food Chain Safety, University of Veterinary Medicine Budapest, 1078 Budapest, Hungary; bali.krisztina@univet.hu (K.B.); kaszab.eszter@univet.hu (E.K.); 3 Department of Biostatistics, University of Veterinary Medicine Budapest, 1078 Budapest, Hungary; fehervari.peter@univet.hu (P.F.); paszerbovics.bettina@univet.hu (B.P.); 4 HUN-REN Veterinary Medical Research Institute, 1143 Budapest, Hungary; 5 One Health Institute, University of Debrecen, 4032 Debrecen, Hungary; 6 Boehringer Ingelheim RCV GmbH & Co KG, Hungarian Branch Office, 1095 Budapest, Hungary; nora.mayer@boehringer-ingelheim.com

**Keywords:** *Dirofilaria immitis*, *Dirofilaria repens*, microfilaria, heartworm disease, dirofilariasis, dogs, Hungary

## Abstract

Heartworm disease—caused by *Dirofilaria immitis*—is an emerging mosquito-borne zoonotic disease with potentially fatal outcomes in dogs. Due to factors such as climate change, animal importation, and animal transport, the prevalence of the infection has increased in countries in Central and Eastern Europe, where the parasite was not detected before. The first autochthonous case of *D. immitis* in dogs in Hungary was reported in 2009. This study assessed the prevalence of *D. immitis* infection and the associated risk factors in domestic dogs in Hungary. A total of 1071 blood samples were collected between March 2023 and February 2024 and were tested using Knott’s test, ELISA, and real-time PCR method. The countrywide prevalence of *D. immitis* was 17.0%, suggesting a continuous increase in the parasite’s spread compared to the previously reported 11.3% prevalence, with age and spending time outdoors being the main risk factors. These results highlight the ongoing need for preventive measures against heartworm disease.

## Simple Summary

Heartworm disease—caused by *Dirofilaria immitis*—is an increasingly common, potentially fatal disease that affects carnivores. In Europe, until the 2000s, heartworm disease occurred only in the Mediterranean regions; however, its prevalence has recently increased in Central and Eastern Europe due to various factors such as climate change, animal importation, and travelling with animals. In Hungary, the first endemic case of *D. immitis* infection in dogs was reported in 2009. This study assessed the prevalence of *D. immitis* infection in domestic dogs in Hungary, and determined risk factors related to the spread of the disease. Based on the countrywide screening of 1071 dogs between March 2023 and February 2024, a prevalence of 17.0% was found, suggesting that the spread of the parasite is still increasing compared to the previously reported 11.3% prevalence. Without appropriate prevention measures, older dogs and animals that spend more time outdoors seem to have a higher risk of becoming infected with the parasite. These results highlight the importance of giving anti-heartworm medication to reduce the spread of *D. immitis*.

## 1. Introduction

*Dirofilaria immitis* (Leidy, 1856) [1] is one of the most dangerous nematode parasites that is transmitted by mosquitoes all over the world as it can cause severe cardiopulmonary disease (commonly known as heartworm disease), mainly in carnivores. *Dirofilaria repens* Railliet & Henry, 1911 [2], is responsible for subcutaneous dirofilariasis and is also widespread, except in the Americas [3]. Several mosquito species can act as vectors of both *D. immitis* and *D. repens*, including *Aedes* spp., *Anopheles* spp., *Culex* spp., and *Ochlerotatus* spp. *D. repens* may also be transmitted by *Coquillettidia* spp. and *Mansonia* spp. [4]. Both parasites are zoonotic: *D. immitis* causes cardiopulmonary symptoms, while *D. repens* causes subcutaneous and ocular symptoms in humans [5,6]. Humans can get infected with *Dirofilaria* spp. similarly to carnivores, i.e., by the bite of mosquito vectors, but they are not suitable hosts: the parasite usually remains in immature form in them without producing microfilaria; thus, humans are considered as dead-end hosts for *Dirofilaria* spp. [7,8,9]. The most common host for both *Dirofilaria* spp. is the domestic dog, but *Dirofilaria* can also infect wild carnivores such as golden jackals and red foxes [10]. Until the 2000s, heartworm disease occurred in Europe only around the Mediterranean—in Greece, Italy, France, Spain, and Portugal [6,11]. A recent study has shown that it has become more prevalent in the Mediterranean region, while also spreading into Central and Eastern Europe [5].

Two main factors are behind the spread of *D. immitis*. Firstly, climate change is allowing vectors that are responsible for transmitting heartworm disease to spread into Central Europe. Rising temperatures are lengthening the breeding season of mosquitos, which is leading to a longer period of exposure each year [12]. In addition to that, the parasite has been introduced to Nordic countries by travelers with pets and the importation of pet dogs [13].

The first autochthonous case of *D. immitis* infection in Hungary was reported in 2009 [14]. Subsequently, several studies carried out in Hungary on wild carnivores and domestic dogs have reported an increasing number of positive cases [6,10,15]. Tolnai et al. [15] reported autochthonous heartworm infections in two dogs, twenty red foxes, and two golden jackals. Bacsadi et al. [10] observed an increase from 0.0% to 2.7% of *D. immitis* infection rates in domestic dogs between 2001 and 2015. The highest prevalence of 11.3% was detected by Farkas et al. [6] in 2017.

The aim of this study was to analyze the latest findings on the prevalence of *D. immitis* infection in domestic dogs in Hungary, and to examine correlations between infection and potential risk factors such as geographical location, neutering status, and the animal’s age.

## 2. Materials and Methods

### 2.1. Sampling Strategy

Between March 2023 and February 2024, blood samples from a total of 1071 dogs were collected and tested for *D. immitis*. The sample size was calculated according to the equation $SE=\sqrt{\frac{p*(1-p)}{n}}$, where the prevalence of infection (p) was estimated as 12.0% based on the latest literature data [6], and a standard error (SE) of 0.01 was allowed. The samples were collected from all 19 counties in Hungary and the number of samples from each county was proportional to the dog population, which was estimated based on the number of registered rabies vaccinations per county (data obtained from the National Food Chain Safety Office of Hungary). The study included only client-owned dogs that visited veterinary clinics throughout the study period. The participation of any veterinary clinic was allowed in the study up to the predetermined maximum sample size per county, considering the inclusion and exclusion criteria. The number of dogs enrolled per county is summarized in Table 1.

### 2.2. Inclusion and Exclusion Criteria

Only dogs that were born in Hungary, had never left the country, and were at least 12 months old were included in this study. In addition, an important inclusion criterion was that the dog must not have received preventive treatment for heartworm disease with macrocyclic lactones in the past year. If any of these criteria were not met, the dog was not included in the study. The owners of all dogs were informed about the study’s terms and conditions, and signed an informed consent form, allowing their animals to participate in the study. For each animal, the following data were recorded: the name of the animal, name of the owner, breed, date of birth, sex, medical history, and date of sampling.

### 2.3. Sampling and Laboratory Analysis

From each dog, 2 mL of venous blood was collected in Vacutainer EDTA (ethylenediaminetetraacetic acid) tubes (BD, Franklin Lakes, NJ, USA) and 1 mL was collected in Vacutainer serum tubes (BD, Franklin Lakes, NJ, USA), and then both samples were transported to an external veterinary diagnostic laboratory (CordenVet, Budapest, Hungary). The samples were first subjected to modified Knott’s test (to detect microfilariae) and ELISA (DiroCHEK, Zoetis, Parsippany, NJ, USA; to detect *D. immitis* antigens), and then to real-time polymerase chain reaction (PCR) analysis (to detect microfilariae and distinguish between *D. immitis* and *D. repens*).

For the modified Knott’s test, 1 mL blood (collected in EDTA tubes) was mixed with 9 mL 2% formalin and then centrifugated for 5 min at 1200 rpm. After removing the supernatant, the sediment was mixed with one drop of 0.1% methylene blue, and then examined as a wet mount [16].

The ELISA test was performed according to the instructions of the manufacturer (DiroCHEK, Zoetis, Parsippany, NJ, USA). From each blood sample (collected in serum tubes), 50 μL was added to microplates coated with antibodies directed against *D. immitis* antigen. Positive and negative control samples included in the DiroCHEK kit were also added to the microplate. This was followed by the addition of 1 drop of the conjugate (antibody labeled with horseradish peroxidase enzyme, also included in the kit), incubation at 21–25 °C for 10 min, and washing five times. The last step was the addition of 2 drops of the chromogenic substrate buffer (also included in the kit), incubation at 21–25 °C for 5 min, and the evaluation of the results. The development of a blue color indicated the presence of *D. immitis* antigens.

For the real-time PCR analysis, blood samples were centrifuged at 1500 rpm for 5 min. Total DNA was purified directly from the blood samples using the DNeasy Blood & Tissue Kit (Qiagen, Hilden, Germany). For discrimination between *D. immitis* and *D. repens*, real-time PCR targeting the cytochrome oxidase subunit I (COI) was performed. The total reaction volume of the 20 μL real-time PCR experiment contained 10 μL master mix Xceed qPCR Probe mix No-ROX (Institute of Applied Biotechnologies, Prague, Czech Republic), 6 μL distilled water, 0.8 μL (10 μM) of each primer, 0.4 μL (10 μM) probe, and 2 μL DNA template. The amplification program included one initial hold at 95 °C for 2 min, followed by 40 cycles consisting of 95 °C for 5 s and 60 °C for 30 s. Real-time PCR was performed on the thermal cycler Rotor-Gene Q (Qiagen, Hilden, Germany). The primer and probe sets designed by Tahir et al. [17] were used to amplify the COI of *D. immitis* and *D. repens*.

### 2.4. Data Analysis

#### 2.4.1. Model Assumptions

Three diagnostic tests were used in the study: modified Knott’s test, ELISA, and real-time PCR with differentiation between *D. immitis* and *D. repens*. To determine the overall prevalence of *D. immitis*, a dog was classified as infected if it yielded a positive result on either ELISA or PCR specific to *D. immitis*. Samples were not subjected to PCR analysis when both the modified Knott’s test and ELISA yielded negative results, as PCR was assumed to be negative in these cases.

As the sensitivity of ELISA is considered to be less than 100%, the possibility of false negative results and, thus, the underestimation of prevalence needed to be investigated. Three potential scenarios arose in cases of negative ELISA results when the possibility of prevalence underestimation was considered:

1. Knott’s test: negative, ELISA: negative, *D. immitis*-specific PCR: negative.
2. Knott’s test: positive, ELISA: negative, *D. immitis*-specific PCR: negative.
3. Knott’s test: positive, ELISA: negative, *D. immitis*-specific PCR: positive.

In the first scenario, it was assumed that if the Knott’s test was negative, the ELISA-negative result was a true negative. All samples that showed the second scenario were PCR positive for *D. repens*, and PCR negative for *D. immitis*. Therefore, the ELISA-negative result in this case was also considered to be a true negative. In the third scenario, even if ELISA produced a false negative result, prevalence was not underestimated, as the sample was categorized as positive based on the positive PCR result. Therefore, it was assumed that the prevalence was not underestimated in this study.

#### 2.4.2. Statistical Evaluation

Data analysis was conducted using R software (version 4.3.2, released 31 October 2023, ucrt). Prevalence estimates for *D. immitis* and their exact 95% confidence intervals were calculated using the epi.conf() function from the epiR package. For geographic reference, the registered addresses of the reporting clinicians were used. These addresses were subsequently matched with settlement type data sourced from OpenStreetMap (OSM), enabling categorization into city, town, and village. In Hungary, places are generally categorized in OpenStreetMap based on population size:

- cities typically have a population exceeding 100,000,
- towns fall within the range of 10,000 to 100,000,
- villages usually have fewer than 10,000 inhabitants.

However, historical significance, administrative status, and local conventions may also influence this categorization. In some cases, a place may be classified as a city due to its historical or administrative importance, even if its population would typically categorize it as a town.

The probability of a positive sample result was modelled using logistic regression, as the outcome is a binary variable with two possible results: positive or negative. The explanatory variables were place, sterilization status, and dog age. Place was categorized into three levels of urbanization (city, town, and village), based on the criteria outlined above. Sterilization status had two levels (sterilized or not), and dog age was treated as a continuous variable. This model allowed us to assess whether the probability of a positive sample result was influenced by place, sterilization status, and dog age. The modelling was performed using R software (version 4.3.2, released 31 October 2023, ucrt), with the stats package and the glm() function. To compare the three levels of place with each other (pairwise comparison), the emmeans package was utilized, along with the emmeans() and pairs() functions. The *p*-value adjustment during pairwise comparisons was performed using the Tukey method.

## 3. Results

### 3.1. Overall Prevalence of Dirofilaria spp.

In total, 1071 blood samples from dogs were examined in this study. From this number, 834 samples were negative for both Knott’s and ELISA tests and were, therefore, assumed to be PCR-negative as well. Among the remaining 237 samples, 182 were classified as *D. immitis*-positive (including mixed infections), as either ELISA or *D. immitis*-specific PCR yielded positive results. The overall estimated prevalence of *D. immitis* was, therefore, 182/1071 (17.0%), with an exact confidence interval of 14.8–19.4%. A total of 109 samples were classified as *D. repens* positive (including mixed infections), based on yielding positive results for *D. repens*-specific PCR. Thus, the prevalence of *D. repens* could be determined as 109/1071 (10.2%), although this was not the main aim of the study. Mixed infection was detected in a total of 54 samples (5.04% of all) that tested positive for both *D. immitis* (by ELISA or PCR) and *D. repens* (by PCR). The results are presented in more detail in Table 2.

### 3.2. County-Specific Prevalence of D. immitis

**Figure 1:**
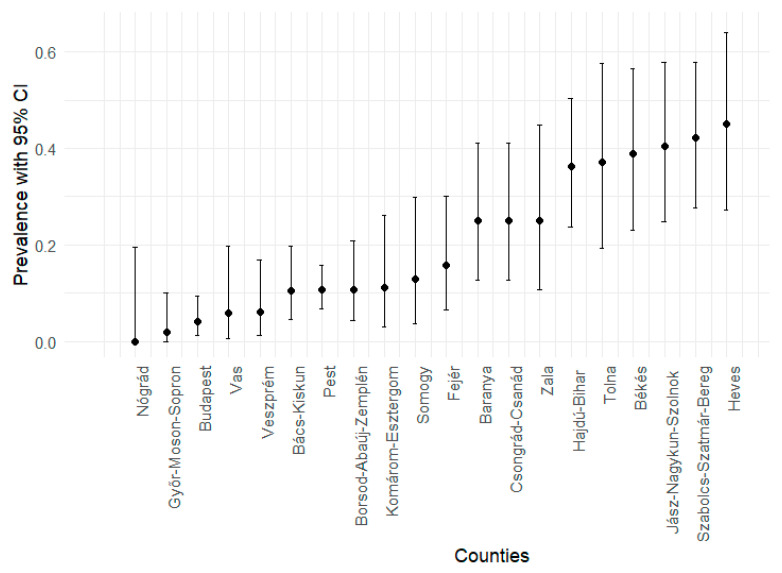
Estimated prevalence rates with 95% confidence intervals across individual counties.

The positive cases for each county were determined using the same criteria as those applied for the overall prevalence. Similarly to the overall prevalence calculations, the prevalence of *D. immitis* and the corresponding 95% confidence intervals were estimated for each county. The results are displayed in Figure 1.

**Figure 2:**
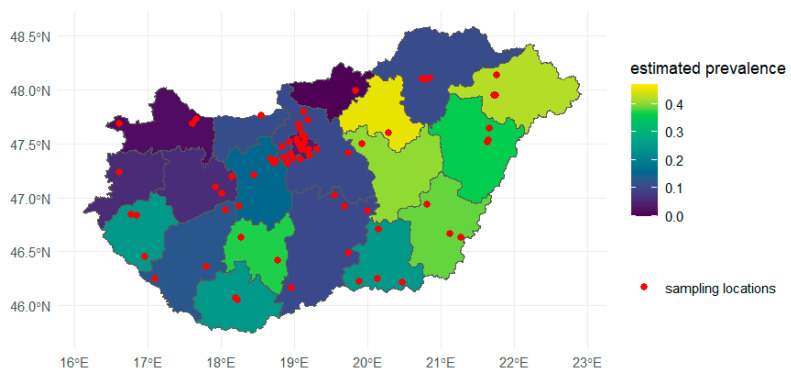
Geographic distribution of *D. immitis* prevalence and corresponding sampling locations.

The geographic distribution of the prevalence of *D. immitis* and the sampling locations are shown in Figure 2.

### 3.3. Logistic Regression Model for Positive Sample Results

From the results of the logistic regression, the coefficients can be used to calculate the odds ratios (OR).

#### 3.3.1. Effect of Urbanization

The results of the logistic regression for the effect of urbanization (city, town, village) on the predicted probabilities of positive sample results can be seen in Table 3.

The odds ratios can be interpreted as follows:

- For the city/town comparison, the odds ratio is 0.70. This means that the odds of a positive result in a city are 0.70 times lower than in a town.
- For the city/village comparison, the odds ratio is 0.54. This indicates that the odds of a positive result in a city are 0.54 times lower than in a village.
- For the town/village comparison, the odds ratio is 0.77. This suggests that the odds of a positive result in a town are 0.77 times lower than in a village.

**Figure 3:**
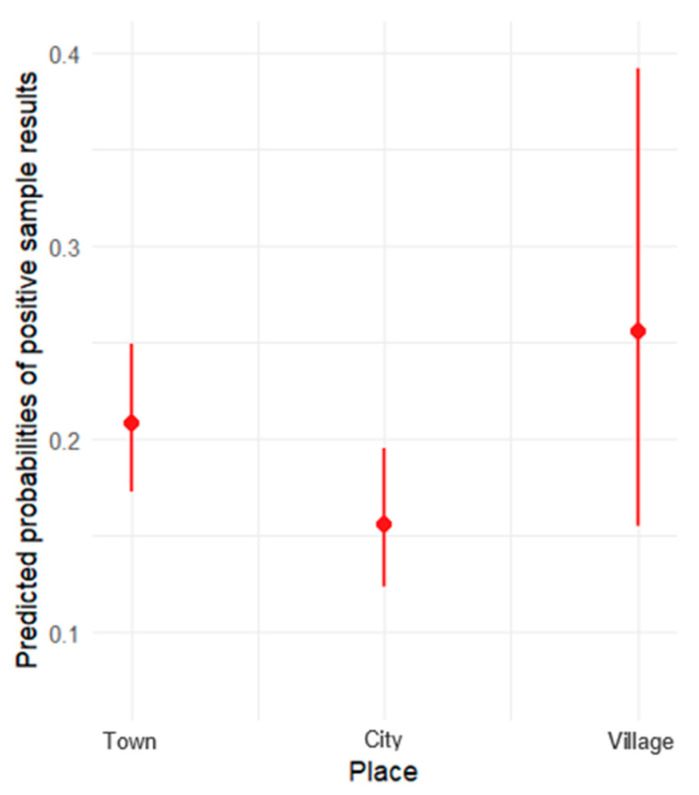
Predicted probabilities of positive sample results by place (city, town, village), with 95% confidence intervals.

Based on the results above, none of the differences were significant at the 5% significance level. The results above are visualized in Figure 3, where the *y*-axis represents the predicted probabilities of positive sample results, and the *x*-axis displays the three place categories: town, village, and city.

#### 3.3.2. Effect of Sterilization Status

The results of the logistic regression for the effect of sterilization status (neutered or not) indicate that the odds of a positive result in the intact group are 1.9 times higher than in the neutered group (standard error: 0.40). The *p*-value (0.0026) suggests that this difference is statistically significant.

**Figure 4:**
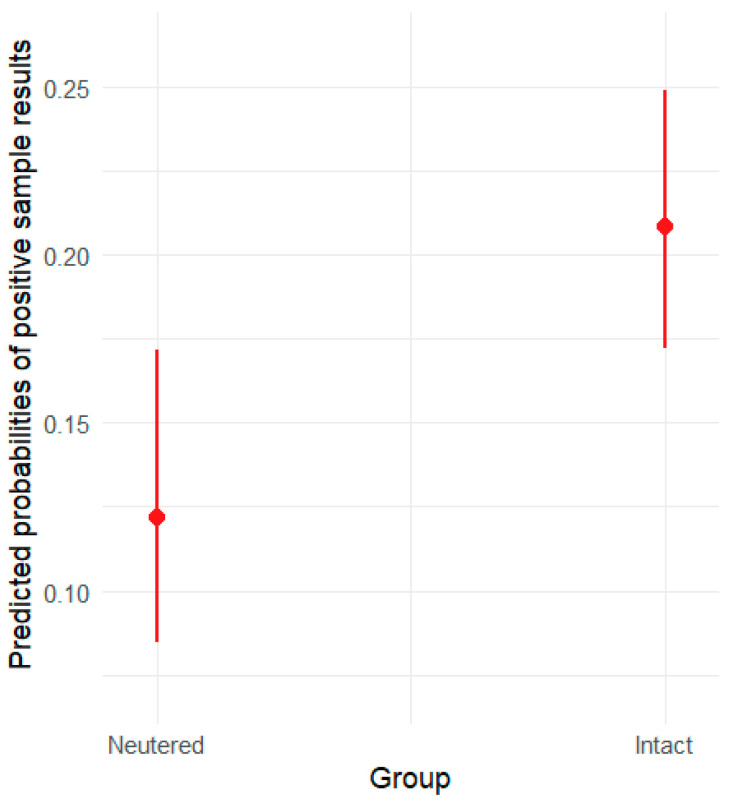
Predicted probabilities of positive sample results by sterilization status (intact, neutered), with 95% confidence intervals.

The results above are visualized in Figure 4, where the y-axis represents the predicted probabilities of positive sample results, and the x-axis displays the two groups: intact and neutered.

#### 3.3.3. Effect of Age

The odds ratio for age was found to be 1.057. Since age is considered as a continuous variable in the model, this means that for each additional year of age, the odds of the sample being positive increase by 5.7%. Furthermore, the effect of age was statistically significant, with a *p*-value of 0.0027.

**Figure 5:**
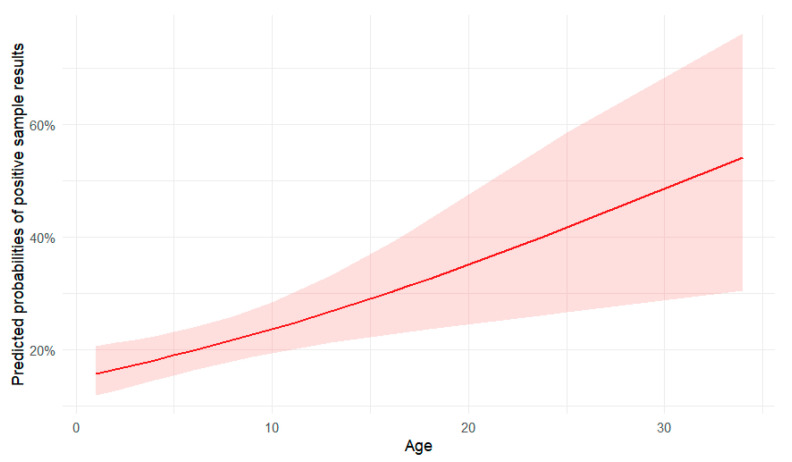
Predicted probabilities of positive sample results by age, with 95% confidence bands.

In Figure 5, the changes in the probability of positive sample results over the years can be observed. The confidence band becomes notably wider towards the upper age range, since there were only 19 dogs aged over 15 years, leading to less certainty in the predictions for older ages.

## 4. Discussion

Until the end of the last century, Hungary was free from *D. immitis*; only imported cases occurred. The first case of autochthonous heartworm infection in dogs was described in 2009 [14], and since then the parasite has spread continuously. A retrospective study based on 2622 autopsied dogs [10] determined the prevalence of *D. immitis* in Hungary as 0.0% between 2001 and 2005, 0.7% between 2006 and 2010, and finally, 2.7% between 2011 and 2015. In 2017, another survey in Hungary with 344 dogs found a heartworm infection rate of 11.3% [6]. In the current study, using 1071 samples, the *D. immitis* prevalence rate was shown to be 17.0%, suggesting that the occurrence and spread of the parasite is still increasing and, thus, highlighting the importance of preventive measures.

Morchón et al. [5] summarized the epidemiological studies of heartworm disease in Europe from 2012 to 2021, noting that the disease had spread into eastern European countries, and that its prevalence continued to increase. The current northernmost autochthonous cases of *D. immitis* infection in the European Union were recently reported in Estonia [18].

Comparing Hungary to neighboring countries with similar climates reveals contrasting prevalence. The prevalence rates of *D. immitis* infection have been recorded at between 1.6 and 3.4% in Slovakia, between 0.46 and 0.6% in Croatia, between 12.7 and 33.3% in Serbia, and from 3.3% to 42.2% with an average of 8.9% in Romania. Recent studies from Austria have mainly reported *D. immitis* infections in dogs that originated from different countries; nevertheless, there is a risk of endemisation in this country too [5]. Recent studies from Portugal and Spain have found prevalence rates of 5.9% and 6.5%, respectively [19,20]. In contrast to these values, the prevalence of *D. immitis* infection in dogs was reported to be 28.6% in Thace, a region that is considered hyperenzootic in Greece [21].

The increasing prevalence of *D. immitis* infection in Europe, including Hungary, can be explained by the effects of climate change and the consequential abundance of the mosquito vectors that allow the development and transmission of heartworms. Furthermore, urbanization and the increased movement of dogs from endemic regions can also contribute to the spread of the parasite [5].

In the current study, no significant differences were found in the probability of heartworm infection when comparing the type of conurbation (city, town, village). In contrast to this finding, previous studies have shown that urbanization is beneficial to the spread of the parasite [5]. On the other hand, increased uptake of heartworm prevention by dog owners in cities, different housing conditions (e.g., living in flats, spending less time outdoors), and more readily available veterinary services may reduce the risk of infection in larger cities. Indeed, Esteves-Guimaraes et al. [19], Montoya-Alonso et al. [20], and Dimzas et al. [21] found that keeping dogs outdoors is a risk factor for *D. immitis* infection.

The analysis of this study’s results showed that the sterilization status of a dog also influences the odds of positive results for *D. immitis* infection. The analysis showed that in intact dogs, the odds ratio of positive sample results is 1.9 times higher than in neutered animals. This may be explained by the fact that dogs—especially males—often roam more during heat periods, meaning that they spend more time outdoors, where infection can occur. This agrees with the findings of Capelli et al. [8], who found that the prevalence of dirofilariasis is higher in male dogs. This assumption is supported by studies that found a significantly higher risk of *D. immitis* in dogs kept outdoors than indoors [19,20,21]. Cuervo et al. [22] suggest that the significant association between gender and dirofilariasis in dogs is likely circumstantial and can be explained by the time spent outdoors. On the other hand, other studies did not find a significant correlation between gender and *D. immitis* infection risk [19,20].

Furthermore, our analysis showed that the probability of *D. immitis* infection increases with age by 5.7% a year. Numerous studies describe a positive correlation between old age and risk of dirofilariasis [6,19,20,21,22,23,24,25], which can be explained by the fact that the longer an animal lives, the greater the period of potential exposure to the mosquito vectors [20,21,22,25].

There is an overlap between the endemic areas of *D. immitis* and *D. repens*, but the latter has also been found in northern and eastern parts of Europe, such as Poland, Lithuania, Ukraine, and Russia [26]. In Hungary the first case of autochthonous *D. repens* infection was confirmed in 1998 [27], although the first national epidemiological study was only carried out two decades later [6]. The prevalence rate of *D. repens* was first determined to be 14.0% [28], which was confirmed (14.2%) by a later study that included 344 dogs [6]. In the current study, a lower prevalence rate of *D. repens* (10.2%) was found. Mixed infection with *D. immitis* and *D. repens* was detected in a total of 54 samples (5.04% overall). However, the exact occurrence of *D. repens* in Hungary should be determined with further studies.

When interpreting the results of this study, the reliability of diagnostic tests should be considered. For the statistical analysis, it was assumed that the PCR test has 100% sensitivity and specificity, eliminating the possibility of false negative or false positive results. The sensitivity and specificity of the primers and probes used for the PCR analysis in this study were confirmed through experimental and in silico analysis by Tahir et al. [17], and the assay was shown to be capable of differentiating between *D. immitis*, *D. repens*, and other filarioid nematodes with 100% coverage. On the other hand, ELISA was assumed to have 100% specificity, ensuring no false positive results; however, its sensitivity was not considered to be 100% [29] as it may be influenced by various factors [30].

When choosing diagnostic methods for the detection of *D. immitis*, the special characteristics of the infection and the limitations of different tests should also be considered. Factors such as long prepatent infections, microfilarial periodicity, same-sex infections, anthelmintic-induced adult sterility, and cases when microfilariae have been destroyed by anthelmintics or by the animal’s immune system may result in lower sensitivity in microfilaria-based diagnostic tests [26]. Commercially available immunochromatographic tests can detect *D. immitis* infections if at least one female worm is present; thus, they have generally high sensitivity and are almost 100% accurate [26]. On the other hand, it is also noteworthy that infected animals might be antigen-negative and microfilaria-positive in case of the death of adult worms with persistence of microfilariae, the transfusion or transplacental transmission of microfilaremic blood, or the presence of blocked antigens (immune complexes). In addition, the antigen is not detectable until 6 months post-infection, and animals may also be antigen negative when only immature worms are present, or when only male worms occur without females [30]. Considering these limiting factors, the combined use of diagnostic tests for adult worm antigens and for microfilariae could be recommended for certain diagnoses.

A recent study has described five cases of human pulmonary dirofilariasis caused by *D. immitis* that were diagnosed over a 12-year period in Hungary [31], which highlights the need to tackle *D. immitis* infections with a One Health approach considering its zoonotic potential. Dimzas et al. [21] also confirmed the risk of human *D. immitis* infections in a hyperenzootic region of Greece, where the proportion of human infections was found to be 23.4% of the corresponding canine cases. Therefore, there is a need for measures to reduce the spread of the parasite, not only for the sake of animal health, but also considering its public health implications.

One of the limitations of the current study is that the registered addresses of the veterinary clinics were used for geographic reference, though they might differ in some cases from the actual location of each dog. As only healthy dogs that were taken to veterinary clinics for regular control examination or vaccination were included in this study, it was assumed that they were examined and sampled in the veterinary clinic closest to their residence. Consequently, the impact of the location difference on the study results was assumed to be minimal. Another limitation is the low number of samples from some counties. Testing a higher number of samples from those counties could have allowed us to find significant differences in the probability of heartworm infection when comparing the types of conurbation and the different regions of Hungary.

## 5. Conclusions

Over the last few decades, the prevalence of *D. immitis* has been increasing all over the world, including in Hungary. Aside from climate change, other predisposing factors can influence the chance of infection in dogs. This study confirms that the age and the neutering of animals can affect the prevalence of heartworm disease, as well as the time spent outdoors where animals are more exposed to infections. In order to better understand the risk factors associated with the spread of the parasite, further examinations are necessary. Monitoring the trends of the parasite’s distribution can lead to a more reliable overview of the epidemiology of *D. immitis*, which is essential for planning country-specific management and protection measures against heartworm disease. The results of this study highlight the continuous need for preventive measures to reduce the spread of the parasite.

## Figures and Tables

**Table 1:** Number of dogs enrolled in the study for each county in Hungary.

County	Sample Number	Proportion of All Samples
Bács-Kiskun	76	7.1%
Baranya	40	3.7%
Békés	36	3.4%
Borsod-Abaúj-Zemplén	65	6.1%
Csongrád-Csanád	40	3.7%
Fejér	44	4.1%
Győr-Moson-Sopron	53	4.9%
Hajdú-Bihar	55	5.1%
Heves	31	2.9%
Jász-Nagykun-Szolnok	37	3.5%
Komárom-Esztergom	36	3.4%
Nógrád	17	1.6%
Pest and Budapest	327	30.5%
Somogy	31	2.9%
Szabolcs-Szatmár-Bereg	45	4.2%
Tolna	27	2.5%
Vas	34	3.2%
Veszprém	49	4.6%
Zala	28	2.6%
Total	1071	100.0%

**Table 2:** Number of samples with different test results and their interpretation regarding *Dirofilaria immitis* and *Dirofilaria repens* positivity.

Test Results					Number of Samples
Knott’s Test	ELISA Test	PCR *Dirofilaria immitis*	PCR *Dirofilaria repens*	Interpretation	
Negative	Negative	Assumed negative	Assumed negative	Negative for both parasites	834
					
Positive	Positive	Positive	Negative	*D. immitis* positive, *D. repens* negative	51
Positive	Negative	Positive	Negative	*D. immitis* positive, *D. repens* negative	11
Negative	Positive	Positive	Negative	*D. immitis* positive, *D. repens* negative	23
Negative	Positive	Negative	Negative	*D. immitis* positive, *D. repens* negative	43
					
Positive	Positive	Positive	Positive	Mixed infection—both *D. immitis* and *D. repens* positive	34
Positive	Positive	Negative	Positive	Mixed infection—both *D. immitis* and *D. repens* positive	11
Positive	Negative	Positive	Positive	Mixed infection—both *D. immitis* and *D. repens* positive	5
Negative	Positive	Positive	Positive	Mixed infection—both *D. immitis* and *D. repens* positive	3
Negative	Positive	Negative	Positive	Mixed infection—both *D. immitis* and *D. repens* positive	1
					
Positive	Negative	Negative	Positive	*D. repens* positive, *D. immitis* negative	55
					
Total—all samples					1071
Total—negative (negative result for both *D. immitis* and *D. repens*)					834
Total—*D. immitis* positive (including 128 samples with *D. immitis* only and 54 samples with mixed infection)					182
Total—*D. repens* positive (including 55 samples with *D. repens* only and 54 samples with mixed infection)					109

**Table 3:** Pairwise comparisons of odds ratios (OR) for positive sample results across different place categories (city, town, village). Odds ratios are presented along with standard errors (SE) and *p*-values.

Contrast	Odds Ratio	SE	*p*-Value
City/town	0.70	0.12	0.1058
City/village	0.54	0.19	0.1708
Town/village	0.77	0.26	0.7099

## Data Availability

The original contributions presented in this study are included in the article. Further inquiries can be directed towards the corresponding author.

## References

[1] Leidy J. (1856). Worms in heart of a dog. Proc. Acad. Nat. Sci. USA.

[2] Railliet A., Henry A. (1911). Sur une filaire péritoneale des porcins. Bull. Soc. Pathol. Exot..

[3] Dantas-Torres F., Otranto D. (2013). Dirofilariosis in the Americas: A more virulent *Dirofilaria immitis*?. Parasites Vectors.

[4] Perles L., Dantas-Torres F., Krücken J., Morchón R., Walochnik J., Otranto D. (2024). Zoonotic dirofilariases: One, no one, or more than one parasite. Trends Parasitol..

[5] Morchón R., Montoya-Alonso J.A., Rodríguez-Escolar I., Carretón E. (2022). What Has Happened to Heartworm Disease in Europe in the Last 10 Years?. Pathogens.

[6] Farkas R., Mag V., Gyurkovszky M., Takács N., Vörös K., Solymosi N. (2020). The current situation of canine dirofilariosis in Hungary. Parasitol. Res..

[7] Simón F., Siles-Lucas M., Morchón R., González-Miguel J., Mellado I., Carretón E., Montoya-Alonso J.A. (2012). Human and animal dirofilariasis: The emergence of a zoonotic mosaic. Clin. Microbiol. Rev..

[8] Capelli G., Genchi C., Baneth G., Bourdeau P., Brianti E., Cardoso L., Danesi P., Fuehrer H.P., Giannelli A., Ionică A.M. (2018). Recent advances on *Dirofilaria repens* in dogs and humans in Europe. Parasites Vectors.

[9] Mendoza-Roldan J.A., Gabrielli S., Cascio A., Manoj R.R.S., Bezerra-Santos M.A., Benelli G., Brianti E., Latrofa M.S., Otranto D. (2021). Zoonotic *Dirofilaria immitis* and *Dirofilaria repens* infection in humans and an integrative approach to the diagnosis. Acta Trop..

[10] Bacsadi Á., Papp A., Szeredi L., Tóth G., Nemes C., Imre V., Tolnai Z., Széll Z., Sréter T. (2016). Retrospective study on the distribution of *Dirofilaria immitis* in dogs in Hungary. Vet. Parasitol..

[11] Genchi C., Rinaldi L., Mortarino M., Genchi M., Cringoli G. (2009). Climate and *Dirofilaria* infection in Europe. Vet. Parasitol..

[12] Fuehrer H.P., Morelli S., Unterköfler M.S., Bajer A., Bakran-Lebl K., Dwużnik-Szarek D., Farkas R., Grandi G., Heddergott M., Jokelainen P. (2021). *Dirofilaria* spp. and *Angiostrongylus vasorum*: Current Risk of Spreading in Central and Northern Europe. Pathogens.

[13] Tiškina V., Jokelainen P. (2017). Vector-borne parasitic infections in dogs in the Baltic and Nordic countries: A questionnaire study to veterinarians on canine babesiosis and infections with *Dirofilaria immitis* and *Dirofilaria repens*. Vet. Parasitol..

[14] Jacsó O., Mándoki M., Majoros G., Pétsch M., Mortarino M., Genchi C., Fok É. (2009). First autochthonous *Dirofilaria immitis* (Leidy, 1856) infection in a dog in Hungary. Helminthologia.

[15] Tolnai Z., Széll Z., Sproch Á., Szeredi L., Sréter T. (2014). *Dirofilaria immitis*: An emerging parasite in dogs, red foxes and golden jackals in Hungary. Vet. Parasitol..

[16] Zajac A., Conboy G. (2012). Veterinary Clinical Parasitology.

[17] Tahir D., Bittar F., Barré-Cardi H., Sow D., Dahmani M., Mediannikov O., Raoult D., Davoust B., Parola P. (2017). Molecular survey of *Dirofilaria immitis* and *Dirofilaria repens* by new real-time TaqMan^®^ PCR assay in dogs and mosquitoes (Diptera: Culicidae) in Corsica (France). Vet. Parasitol..

[18] Mõttus M., Mõtsküla P.F., Jokelainen P. (2024). Heartworm disease in domestic dogs in Estonia: Indication of local circulation of the zoonotic parasite *Dirofilaria immitis* farther north than previously reported. Parasites Vectors.

[19] Esteves-Guimarães J., Matos J.I., Leal-Sousa B., Oliveira P., Lobo L., Silvestre-Ferreira A.C., Soares C.S., Rodríguez-Escolar I., Carretón E., Morchón R. (2024). Current State of Canine Heartworm in Portugal. Animals.

[20] Montoya-Alonso J.A., Morchón R., García-Rodríguez S.N., Falcón-Cordón Y., Costa-Rodríguez N., Matos J.I., Rodríguez Escolar I., Carretón E. (2022). Expansion of Canine Heartworm in Spain. Animals.

[21] Dimzas D., Aindelis G., Tamvakis A., Chatzoudi S., Chlichlia K., Panopoulou M., Diakou A. (2024). *Dirofilaria immitis* and *Dirofilaria repens*: Investigating the Prevalence of Zoonotic Parasites in Dogs and Humans in a Hyperenzootic Area. Animals.

[22] Cuervo P.F., Di Cataldo S., Fantozzi M.C., Rodríguez M.B., Pedrosa A., Mera Y., Sierra R. (2024). Host drivers of canine dirofilariosis in an arid environment of western Argentina. Parasitol. Res..

[23] Fioretti D.P., Diaferia M., Grelloni V., Maresca C. (2003). Canine filariosis in Umbria: An update of the occurrence one year after the first observation of autochthonous foci. Parassitologia.

[24] Tasić A., Rossi L., Tasić S., Miladinović-Tasić N., Ilić T., Dimitrijević S. (2008). Survey of canine dirofilariasis in Vojvodina, Serbia. Parasitol. Res..

[25] Iglódyová A., Miterpáková M., Hurníková Z., Antolová D., Dubinský P., Letková V. (2012). Canine dirofilariosis under specific environmental conditions of the Eastern Slovak Lowland. Ann. Agric. Environ. Med..

[26] Fehr J.E., Schnyder M., Joekel D.E., Pantchev N., Sarkunas M., Torgerson P., Deplazes P. (2022). Estimated specific antibody-based true sero-prevalences of canine filariosis in dogs in Central Europe and the UK. Parasitol. Res..

[27] Fok É., Szabó Z., Farkas R. (1998). The first autochthonous case of a dog infected with *Dirofilaria repens* in Hungary. Kisállatorvoslás.

[28] Fok É., Kiss G., Majoros G., Jacsó O., Farkas R., Gyurkovszky M., Genchi C., Rinaldi L., Cringoli G. (2007). Preliminary results of an epidemiological survey on dirofilariosis of dogs and cats in Hungary. Dirofilaria immitis and D. repens in Dog and Cat and Human Infections.

[29] Carmichael J., McCall S., Dicosty U., Mansour A., Roycroft L. (2017). Evaluation of *Dirofilaria immitis* antigen detection comparing heated and unheated serum in dogs with experimental heartworm infections. Parasites Vectors.

[30] Little S., Saleh M., Wohltjen M., Nagamori Y. (2018). Prime detection of *Dirofilaria immitis*: Understanding the influence of blocked antigen on heartworm test performance. Parasites Vectors.

[31] Kuthi L., Zombori T., Tiszlavicz L., Hegedűs F., Almási S., Baráth B., Almakrami M., Ej M.J., Barta N., Ujfaludi Z. (2024). Emerging human pulmonary dirofilariasis in Hungary: A single center experience. Diagn. Pathol..

